# Supplementary material for: Isoquercitrin Attenuates Osteogenic Injury in MC3T3 Osteoblastic Cells and the Zebrafish Model via the Keap1-Nrf2-ARE Pathway
Source: Molecules. 2022 May 27;27(11):3459. doi: 10.3390/molecules27113459 (PMC9182080; doi:10.3390/molecules27113459)
Supplement: Supplementary file 1 [file molecules-27-03459-s001.zip › molecules-1691787-supplementary.pdf]

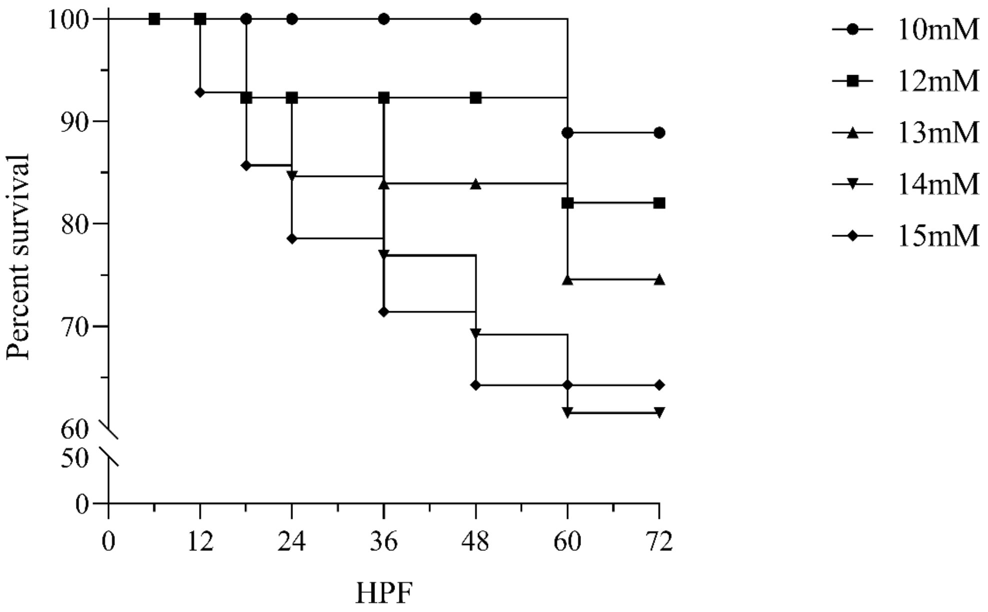

Figure S1: Screening of AAPH concentration in 72hpf zebrafish.

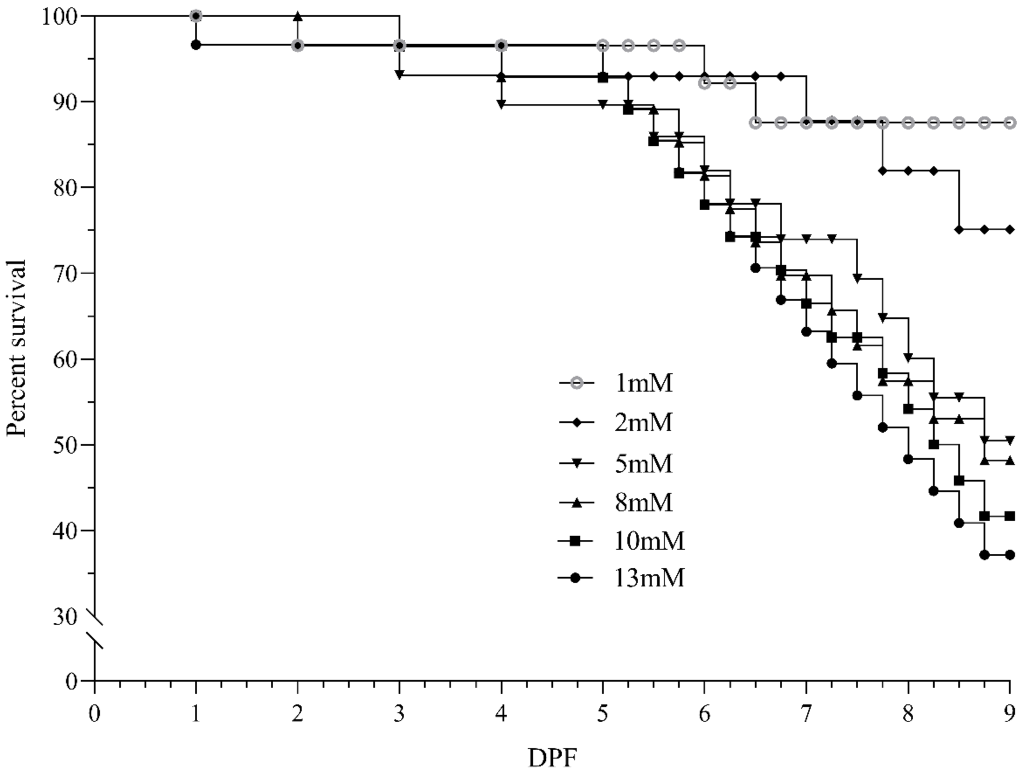

Figure S2: Screening of AAPH concentration in 9dpf zebrafish.
